# Gait and Functional Mobility in Multiple Sclerosis: Immediate Effects of Transcranial Direct Current Stimulation (tDCS) Paired With Aerobic Exercise

**DOI:** 10.3389/fneur.2020.00310

**Published:** 2020-05-05

**Authors:** Giuseppina Pilloni, Claire Choi, Giancarlo Coghe, Eleonora Cocco, Lauren B. Krupp, Massimiliano Pau, Leigh E. Charvet

**Affiliations:** ^1^NYU Langone Health, Department of Neurology, New York, NY, United States; ^2^Department of Mechanical, Chemical and Materials Engineering, University of Cagliari, Cagliari, Italy; ^3^SUNY Downstate, Department of Medicine, New York, NY, United States; ^4^Department of Medical Sciences and Public Health, University of Cagliari, Cagliari, Italy

**Keywords:** transcranial direct current stimulation, tDCS, non-invasive brain stimulation, multiple sclerosis, motor rehabilitation, gait, functional mobility, aerobic exercise

## Abstract

Walking impairments are a debilitating feature of multiple sclerosis (MS) because of the direct interference with daily activity. The management of motor symptoms in those with MS remains a therapeutic challenge. Transcranial direct current stimulation (tDCS) is a type of non-invasive brain stimulation that is emerging as a promising rehabilitative tool but requires further characterization to determine its optimal therapeutic use. In this randomized, sham-controlled proof-of-concept study, we tested the immediate effects of a single tDCS session on walking and functional mobility in those with MS. Seventeen participants with MS completed one 20-min session of aerobic exercise, randomly assigned to be paired with either active (2.5 mA, *n* = 9) or sham (*n* = 8) tDCS over the primary motor cortex (M1). The groups (active vs. sham) were matched according to gender (50% vs. 60% F), age (52.1 ± 12.85 vs. 54.2 ± 8.5 years), and level of neurological disability (median Expanded Disability Status Scale score 5.5 vs. 5). Gait speed on the 10-m walk test and the Timed Up and Go (TUG) time were measured by a wearable inertial sensor immediately before and following the 20-min session, with changes compared between conditions and time. There were no significant differences in gait speed or TUG time changes following the session in the full sample or between the active vs. sham groups. These findings suggest that a single session of anodal tDCS over M1 is not sufficient to affect walking and functional mobility in those with MS. Instead, behavioral motor response of tDCS is likely to be cumulative, and the effects of multiple tDCS sessions require further study.

**Clinical Trial Registration:** www.ClinicalTrials.gov, identifier: NCT03658668.

## Introduction

Multiple sclerosis (MS) is the leading cause of progressive functional impairments in younger adults of working age (1). Multiple sclerosis symptoms are often variable across individuals and can affect motor, sensory, and cognitive functions (2). Loss of mobility is a key concern due to the interference with independence and the ability to complete activities of daily living (3, 4). Multiple factors contribute to the degeneration of MS ambulatory ability, such as muscle weakness, abnormal walking mechanisms, balance problems, spasticity, and fatigue (3, 5). While there is no typical pattern of MS gait disturbance, impairments often include reductions in gait velocity and step length (6, 7). Symptomatic treatment is an important topic for the management of MS (8, 9), with a strong unmet need for non-pharmacologic options to preserve and recover from MS-related walking impairments (10).

Non-invasive brain stimulation techniques, such as transcranial direct current stimulation (tDCS), are being studied for a range of applications in MS symptom management, including fatigue, cognitive deficits, neuropathic pain, and motor impairments (11). tDCS delivers weak electrical currents (1.0–2.5 mA) passed through electrodes (anode and cathode) placed on the scalp targeting brain regions of interest. This technique has been used to modulate the resting membrane potential in cortical and subcortical tissue promoting cell plasticity (12, 13). The neurophysiological response seems to be achieved through mechanisms of long-term potentiation or long-term depression of synapses (14, 15). tDCS can influence neural activity in a polarity-dependent manner: cortical excitability can be increased (under the anode) or reduced (under the cathode) in the underlying cortex (16).

Despite intense recent investigation of tDCS, parameters for dosing in terms of timing of application in relation to a paired training activity, current intensity, duration, and number of sessions remain largely undefined (17, 18). A growing number of studies, albeit with mixed results, have overall demonstrated the efficacy of a single anodal tDCS session over the primary motor cortex (M1) to improve motor performance in both healthy controls and patients with motor disorders (19–22). The probability to detect either neurophysiological or clinical responses still remains unclear, since treatment responses may be achieved by both single or repeated tDCS applications (23–26).

Previous findings have reported mixed effects after the application of tDCS over M1 on motor outcome variables (e.g., mobility and functionality of lower limbs, muscle strength, functional ambulation) in MS patients (27, 28). Given the current intensity delivered by tDCS is too low to generate *de novo* neuronal action potentials (29), the working mechanism is based on the “functional targeting” principle (30), where tDCS facilitates neuronal activation of specific pathways involved during the execution of a paired training activity. Therefore, potential interactions and synergies between tDCS and aerobic exercise have been recently studied to improve the recovery process within neurological conditions or to increase performance (31).

Aerobic exercise has demonstrated benefit in MS, with aerobic training shown to improve gait speed, stride length, and walking distance (32). Transcranial direct current stimulation may interact with exercise training enhancing the acute effect on motor functions and promote long-lasting benefits (31, 33). Thus, the use of tDCS during aerobic exercise may enhance the therapeutic effects via greater activation of neuroplastic mechanisms.

Specific electrode montages have varied across studies aimed at improving motor performance and symptoms to date, but most of them applied the anode over M1 area (34–36). Some studies conceptualized alternative motor electrode montages, varying electrode dimensions, and the position of the cathode, in order to optimize the stimulation of the lower limb motor cortex (37, 38). However, evidence is mixed as to whether these variations can improve effects compared to the standard motor montage (anode over M1 and cathode over the contralateral supraorbital area) [(37, 39)].

The current intensity has also varied across the studies, but 2.5 mA is the higher amperage of current clinical convention across trials (40, 41). Preliminary evidence and theoretical models (42, 43) provide support for the utilization of a relative higher stimulation amperage to increase cortical excitability. Moreover, previous studies have found good tolerability with higher amperage as the improved promotion of optimal and measurable response (44, 45).

The aim of this study was to test the motor response following a single session of tDCS over M1, clarifying its efficacy in enhancing the effect of aerobic exercise on walking and functional mobility performance in MS patients.

## Methods

### Participants

Seventeen participants with either relapsing-remitting MS (RRMS) or secondary progressive MS (SPMS) were recruited. Eligibility criteria included ages 18–70 years, level of neurologic disability as measured by the Expanded Disability Status Scale (EDSS) (46) score from 1.0 to 6.5, and the ability to independently walk (with or without an assistive device) for at least 20 m. Potential participants were excluded if they had any history of brain trauma or seizures, any skin disorder or skin sensitive area near the stimulation locations, or were unable to understand the informed consent process and/or study procedures.

All participants provided written informed consent, and the study was conducted at the MS Comprehensive Care Center, NYU Langone Health. Ethical approval was obtained from the Institutional Review Board Committee of the New York University School of Medicine and followed the Ethical Principles for Medical Research Involving Human Subjects outlined in the Declaration of Helsinki.

### Study Design and Experimental Protocol

This proof-of-concept study is part of a larger and ongoing clinical trial that employs a double-blind, sham-controlled, randomized design of tDCS paired with aerobic exercise. During the baseline visit, participants were screened for eligibility, consented, and randomized to the active or sham arm within strata defined by EDSS level [EDSS “low” (0–3.5) vs. “high” (4.0–6.5) score] and age (18–45 years vs. 46–65 years). To ensure the double-blind nature of the study (both patient and the technician involved in treatment and assessment), an independent technician completed the randomization and pre-programmed the tDCS device in advance to deliver active or sham stimulation accordingly.

### Intervention: tDCS Paired With Aerobic Exercise

For the current analyses, we analyzed gait and functional mobility measures before and after the first tDCS + exercise session. Both the active and sham participants completed 20 min of stimulation during exercise using a recumbent combination arm/leg elliptical ergometer (PhysioStep LTX-700). The exercise period included heart rate (HR) monitoring via Fitbit wristband (Fitbit Inc., California, USA) to ensure that each participant met the recommended target HR for the physical exercise for MS, training at moderate intensity corresponding to 60–80% of age-predicted maximum HR (47).

Transcranial direct current stimulation was applied to the M1 cortex with the goal of enhancing the activation of the cortical pathways involved and activated during pedaling/cycling (48, 49). Active and sham tDCS was delivered using the 1 × 1 tDCS mini-clinical trial device (mini-CT; Soterix Medical Inc., New York, NY, USA) using an optimized motor montage targeting the M1 area with supraorbital exit (C3 anode/Fp2 cathode according to 10/20 EEG), with two pre-saturated sponge surface electrodes (square shape, 5 × 5 cm^2^). The current intensity was set at 2.5 mA, with the goal of increasing cortical excitability to promote optimal and measurable clinical response (42, 43, 45).

All study procedures were the same for active and sham conditions. For the active participants, the current intensity was set at 2.5 mA for the entire session. For the sham participants, and following the current blinding recommendations, the device delivered a 60-s ramp up/down to the 2.5-mA target at the beginning and end of the 20-min period, with no other current delivery. In this way, the sham procedure produces similar sensory experiences to mask the stimulation condition administered (50, 51).

### Motor Assessment, Blinding Assessment, and Motor Outcomes

To measure changes in gait and functional mobility, the instrumented 10-m walking test and the instrumented Timed Up and Go (TUG) test were measured using a single wearable inertial sensor (G-Sensor® BTS Bioengineering S.p.A., Milan, Italy). Both tests were performed twice consecutively, the first time for familiarization, and the second time for data capture. The inertial sensor was positioned to the participant's waist using a semielastic belt (covering the L4–L5 intervertebral disc for walking assessment and L1-L2 for TUG test providing acceleration values along three orthogonal axes and transmitted via Bluetooth to a PC, where the raw accelerations were processed. For the 10-m walking test, participants were instructed to walk along a 10-m path at their typical speed. For the TUG, participants were instructed to sit on a standard armless chair with back support. At start, they stood up, walked for 3 m at self-selected speed, performed a 180° turn around at a cone, and then walked back to the chair, and then performed a second 180° turn to sit down.

Post-processing of the acceleration signals using dedicated software (BTS G-Studio; BTS Bioengineering S.p.A.) allowed computing the following parameters:

- 10-m walking test:
  - the mean velocity of progression (m/s);
- TUG test:
  - TUG time: the time needed to complete the test (s).

Both the 10-m walk test and the TUG are validated as standard clinical tests with high test–retest reliability for measuring walking function and functional mobility in patients with MS (52, 53).

At the end of the study, blinding integrity was assessed by asking the participants to guess the received treatment.

### Statistical Analysis

The collected data were analyzed using the statistical package SPSS version 25 (SPSS, Inc., Chicago, IL, USA). Descriptive analyses were generated for demographic and clinical variables of the two arms. The normal distribution of the dependent variables (gait velocity and TUG time) was assessed by the Kolmogorov–Smirnov test. The dependent variables of the study met the criteria of normality. Because of the normal distribution, a general mixed-model analysis of variance (ANOVA) 2 × 2 (intervention × time) was performed to examine the effect of the between-subjects factor treatment (active, sham) and the within-subjects factor time (pre-assessment and post-assessment). The type I error (α) was set at 0.05, and the effect sizes were assessed using the η^2^ coefficient. When a significant main effect was reached, *post-hoc* tests with Sidak correction for multiple comparisons were conducted to assess treatment or time point differences.

## Results

The groups were matched in terms of demographic and clinical features (50 vs. 60% *F*; median EDSS: 5.5 vs. 5; 2 RRMS, 7 SPMS vs. 3 RRMS, 5 SPMS; age 52.1 ± 12.85 vs. 54.2 ± 8.5 years, active vs. sham respectively, all *p* > 0.05).

Stimulation was well-tolerated across participants and with side effects of itching, tingling, and head pain. No side effect reached an intensity level of >7 (rated on a 0- to 10-point scale) for any participant, and all side effects resolved at the end of the stimulation period.

Overall, the tDCS condition assignment (active, sham) was identified correctly by 28% of participants (specifically, the 33% of the participants in the sham group). The result of the blinding integrity is in agreement with the standards suggested in previous studies (51, 54).

All participants completed the 20-min aerobic exercise maintaining the targeted moderate level. The average HR during the session was 110.9 ± 4.0 beats/min.

**Figure 1:**
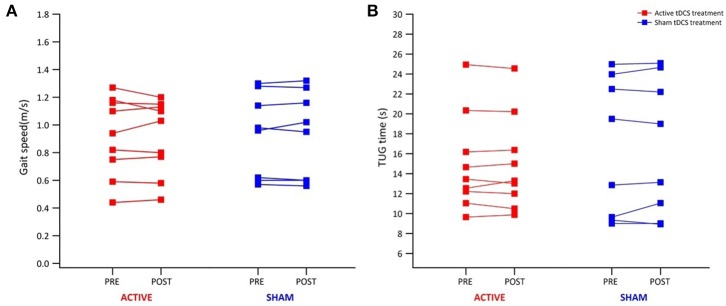
Individual results from 10-m walk test and TUG test for all participants. Individual results of each participant of the active (red) and sham group (blue) before and immediately after tDCS paired with aerobic exercise for gait speed **(A)** and TUG time **(B)**.

**Figure 2:**
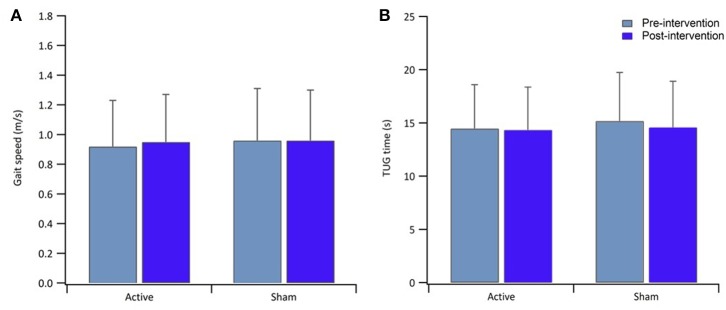
Bar graphs show result of gait speed **(A)** and TUG time **(B)** for the active and sham group, pre-intervention (light blue) and post-intervention (blue). Error bars in both graphs indicate ± SD.

Table 1 reports data of gait velocity and TUG time as mean ± SD. The ANOVA test indicated no significant changes in gait speed and TUG time after one single session in either the whole group or active vs. sham tDCS (Figures 1, 2). There were no significant main effects of the intervention [*F*_(1,15)_ = 0.074, *p* = 0.861, η^2^ = 0.006; *F*_(1,15)_ = 0.087, *p* = 0.883, η^2^ = 0.002], as well as of the time [*F*_(1,15)_ = 2.346, *p* = 0.070, η^2^ = 0.101; *F*_(1,15)_ = 1.784, *p* = 0.239, η^2^ = 0.088] and time × intervention interactions [*F*_(1,15)_ = 1.946, *p* = 0.115, η^2^ = 0.093; *F*_(1,15)_ = 1.381, *p* = 0.446, η^2^ = 0.038] for gait speed and TUG time, respectively. These findings indicate no immediate effect on walking and functional mobility performance with either tDCS paired with aerobic exercise or aerobic exercise alone.

**Table 1:** *Post-hoc* pairwise comparison of gait speed and TUG time pre-intervention and post-intervention for active and sham group.

	**Active group (*****n*** **=** **9)**			**Sham group (*****n*** **=** **8)**		
	**Pre-intervention**	**Post-intervention**	**Comparison pre vs. post *p*-value**	**Pre-intervention**	**Post-intervention**	**Comparison pre vs. post *p*-value**
Gait speed (m/s)	0.92 ± 0.31	0.95 ± 0.32	0.456	0.96 ± 0.35	0.96 ± 0.34	0.558
TUG time (s)	14.48 ± 4.11	14.34 ± 4.02	0.195	15.19 ± 4.56	14.58 ± 4.33	0.103

*Values are reported as mean ± SD*.

## Discussion

The aim of this study was to investigate the immediate effects of a single tDCS session over M1 on the behavioral motor responses of patients with MS. We did not find any significant treatment effect in walking speed and TUG time following the application of a single session of tDCS paired with aerobic exercise. While changes have been reported following a single session in prior studies (19, 25), our findings are consistent with the growing literature across neurological disorders indicating that a single session of tDCS is not enough to lead to meaningful or measurable behavioral outcomes (24, 55) or to enhance the benefits of physical training on motor functions in those with MS.

Previously, the effects of a single tDCS application over M1 on gait in MS (56) during the 6-min walk test (2 mA, 6 min) did not report any improvement in distance walked, gait velocity, and stride length. However, this study was limited by the use of a short duration of the stimulation (6 min) (56), while the present study adopted a longer duration of stimulation equal to 20 min according to recommendations based on evidence to date (40, 41).

Our findings are consistent with another previous report of tDCS and hand functioning (23) in MS, where they explored the effect of a single session of anodal tDCS applied to the M1 contralateral to the affected hand. The authors reported an increase in the corticospinal output and projection strengthening evaluated by using transcranial magnetic stimulation, but no behavioral motor effects were measured (23).

The current results do not necessarily imply the absence of an increase activation of the underlying brain region. An enhancement of MEP amplitudes, corticospinal output, and projection strength has been reported with a single application, even in the absence of measurable behavioral or clinical outcomes (23, 57).

When targeting behavioral outcomes, dosing dimensions such as the specific current intensity, duration of the stimulation, and number of sessions, or response variability remain unknown. It is important to consider pairing tDCS with behavioral training or rehabilitative activity as a critical dimension of dosing (31, 35, 58, 59) to improve motor outcomes. This multimodal approach is likely to have stronger effects on promoting synaptic changes and increasing the likelihood of detecting behavioral motor responses.

Specific to stimulation intensity and duration, findings are mixed (45, 60). We chose the conventional 20 min of stimulation at the higher 2.5mA current intensity under the hypothesis that these parameters would lead to higher brain activation (42); however, tDCS dosing may not necessarily be linear. In fact, either increasing the current intensity >4 mA or prolonging the stimulation more than 30-min duration is not always accompanied by an increase of its efficacy, with either change in the direction potentially leading to different patterns of neuronal activation (61, 62). It may be that alternative M1 (or other) montages as well as stimulation intensity could have resulted in different findings.

Nonetheless, the absence of effect of one tDCS application in MS for motor outcomes is an important finding as many studies continue to evaluate the clinical responses of a single session of tDCS. Recent work in MS (28) indicates that, in MS, multiple stimulation sessions can lead to benefit. In a sample of *n* = 13, those who received anodal tDCS stimulation over the M1 walked faster during the Timed 25-Foot Walk after seven sessions (28). The number of overall applications may be key in evaluating its rehabilitative and restorative potential according to the consolidation effects (63–65).

Limitations of the current study include its relatively small sample size. With a larger sample or greater range of MS participants, it is possible that more subtle effects of an initial tDCS application could be detected. In addition, while one strength of our study is the use of an advance technology to detect and characterize motor outcomes, we were not able to correspond findings to actual neurophysiological measures (e.g., structural and metabolic analysis of brain functions in response to the stimulation). The study would also have been strengthened by including a condition with tDCS only (without exercise) as an additional comparison.

Future studies need to more clearly define the effectiveness of tDCS as treatment option or as therapeutic adjuvant in motor rehabilitation. Clinical studies need to be designed to clarify the dimensions of dosing, not only including number of sessions, current intensity, and electrode montage, but also exploring other dosing dimensions represented by the combination of the practice of motor task or physical training and its timing of application (before/during/after stimulation). It would also be important to integrate the acquisition of functional neuroimaging (e.g., functional magnetic resonance imaging, positron emission tomography) with tDCS, to better understand how the stimulation modulates ongoing brain activity and connectivity.

## Conclusion

Taken together, these findings indicate that a single session of anodal tDCS over M1 is not sufficient to improve walking and functional mobility in MS. Instead, behavioral effects of tDCS are likely to be cumulative, with a single session of tDCS able to provoke neurophysiological changes. Future studies with multiple and repeated sessions and paired with motor training are warranted in order to test the cumulative response in neural excitability outlasting the stimulation period to determine optimal clinical utilization.

## Data Availability Statement

The datasets generated for this study are available to any qualified researcher on request to the corresponding author.

## Ethics Statement

The studies involving human participants were reviewed and approved by the Institutional Review Board Committee of the New York University School of Medicine. Study procedures were conducted in accordance with the Declaration of Helsinki. The patients/participants provided their written informed consent to participate in this study.

## Author Contributions

GP, LC, MP, EC, and GC designed the study. Participants were recruited by CC and GP, and screened by LK. GP performed data collection and analysis. GP and LC interpreted the results. The manuscript was drafted by GP and LC. All the authors critically revised the manuscript and approved the final version.

## Conflict of Interest

The authors declare that the research was conducted in the absence of any commercial or financial relationships that could be construed as a potential conflict of interest.
